# Correlation between heart rate variability and estradiol, progesterone, and the estradiol/progesterone ratio across menstrual phases in healthy women

**DOI:** 10.14814/phy2.70887

**Published:** 2026-04-24

**Authors:** Tara R. Salih, Darya S. Abdulateef

**Affiliations:** ^1^ Basic Medical Sciences Department University of Sulaimani College of Medicine Sulaymaniyah Kurdistan Region Iraq

**Keywords:** autonomic nervous system, estrogen, heart rate variability, menstrual cycle phases, progesterone

## Abstract

Heart rate variability (HRV) is widely used to assess cardiac health, yet uncertainty persists regarding HRV variations across the menstrual cycle and their association with reproductive hormones. Few studies have evaluated the full spectrum of HRV parameters, and most estimated menstrual phases from the last menstrual period without considering individual cycle length. This study aimed to compare HRV parameters across three menstrual phase groups, using accurate cycle tracking based on the next menstrual period and actual cycle length, and to correlate HRV with estradiol, progesterone, and the estradiol/progesterone (E/P) ratio. In 112 healthy women with regular cycles, HRV parameters were measured using a heart rate sensor, and serum hormone levels were obtained. Menstrual phase groups were defined using back‐extrapolation from the next cycle. Data from 99 participants were analyzed: menstruation (*n* = 38), periovulatory (*n* = 30), and premenstrual phase (*n* = 31). HF was significantly higher during the peri‐ovulatory phase, while estradiol and progesterone peaked premenstrually. The E/P ratio was highest pre‐ovulatory. Estradiol correlated with most HRV measures; however, only the mean RR‐interval in the premenstrual phase was statistically significant, with estradiol emerging as a negative independent predictor. These findings conclude notable HRV variation across menstrual phases and suggest that estradiol fluctuations may influence autonomic regulation.

## 1 INTRODUCTION

The menstrual cycle consists of two interrelated components: the ovarian cycle and the uterine cycle. The ovarian cycle includes the follicular phase, characterized by follicular development and increasing estrogen (E2) levels, and the luteal phase, during which the corpus luteum forms and secretes progesterone (P4) following ovulation (Hall, 2025). The uterine cycle consists of the menstrual phase, involving shedding of the endometrial lining; the proliferative phase, during which E2 stimulates endometrial regeneration; and the secretory phase, when P4 prepares the endometrium for potential implantation (Barret et al., 2019; Hall, 2025). The hormonal changes during the menstrual cycle affect more than just reproduction, influencing other body functions such as sleep regulation, circadian rhythms, and autonomic nervous system (ANS) activity. For example, studies have shown a reduction in vagal influence on the heart as the cycle progresses from the follicular to the luteal phase. In addition, baroreflex sensitivity appears to increase during the luteal phase due to higher P4, which stimulates sympathetic nervous activity and the phrenic nerve (Patra et al., 2023). Heart rate tends to drop before ovulation, possibly reflecting reduced sympathetic activity or enhanced vagal influence associated with peak E2 levels or the luteinizing hormone (LH) surge, and is higher during the luteal phase (Biswas & Ghosh, 2024).

The spectral components of heart rate variability (HRV) are one of the most indicative markers of autonomic regulation. Notably high‐frequency power (HF), which reflects parasympathetic (vagal) tone, and low‐frequency power (LF), which, though modulated by both sympathetic and parasympathetic branches, is often considered alongside HF via the LF/HF ratio, which is a measure of vago‐sympathetic balance, and a high level is suggestive of sympathetic activity (Schmalenberger et al., 2019; Shaffer & Ginsberg, 2017; Sharma et al., 2022). A growing body of research indicates that these autonomic markers fluctuate throughout the cycle in response to shifts in ovarian hormones, especially E2 and P4 (Biswas & Ghosh, 2024; Patra et al., 2023; Schmalenberger et al., 2019). There is a reduction of HF over time from menstruation through the late luteal phase, strengthening the hypothesis that HRV is hormonally sensitive, particularly in its vagal components, and indicating a loss of parasympathetic tone at a time when P4 levels are typically elevated (Armbruster et al., 2018). A reduction in HF and a rise in the LF/HF ratio from the follicular to the luteal phase found in studies is parallel with an elevation in both P4 and heart rate (Bai et al., 2009; Leicht et al., 2003; Sato et al., 1995; Tenan et al., 2014; Weissman et al., 2009; Yildirir et al., 2006).

Despite the overall stability, this vagal modulation, mirrored in HF indices, corresponded with E2 fluctuations, adding quite a support to the hormone's cardioprotective potential (Leicht et al., 2003).

A rapid E2 elevation in women undergoing ovulation induction corresponded with a marked increase in HF power, concluding that an acute rise in E2 during ovulation was linked to increased vagal activity and a shift in sympatho‐vagal balance (Weissman et al., 2009), although it is confined to spectral markers with no substantial change observed in time‐domain indices. This is consistent with earlier findings demonstrating positive correlations between E2 levels and HRV markers at ovulation (Leicht et al., 2003).

Although most of the studies associated parasympathetic markers with E2 at the follicular or peri‐ovulatory phase (Biswas & Ghosh, 2024; Leicht et al., 2003), some studies showed either stable ANS measurements across the phases (Leicht et al., 2003) or found an increase in total power and HF in the luteal phase compared to the follicular phase and suggested stronger vagal modulation during luteal stages (Princi et al., 2005). Further investigation revealed that differences in HRV across phases may disappear if HRV is recorded beyond the optimal time frame, specifically during late follicular days instead of early follicular ones, reinforcing the critical role of accurate timing in HRV‐hormone research (Sato et al., 1995). In that regard, there are controversies about the differences in HRV across the menstrual cycle and its correlation with reproductive hormones.

Accurate timing is further complicated by considerable variation in cycle phases, even among healthy, regularly cycling women (Fehring et al., 2006). Because the luteal phase remains relatively stable at approximately 14 days, most variation in total menstrual cycle length reflects changes in the duration of the follicular phase; it ranges between 10 and 16 days (Biswas & Ghosh, 2024), whereas in a 28‐day cycle, the follicular phase spans from the first day of menstruation to ovulation on day 14 (Monis & Tetrokalashvili, 2025). However, ovulation doesn't always occur on day 14; only about 3%–10% of cycles show ovulation on that day (Wilcox et al., 2000).

In that regard, although HRV is widely used as a measure of ANS activity and has been correlated with menstrual cycle phases in previous research, findings regarding HRV changes across the menstrual cycle and their relationship with reproductive hormones remain inconsistent. Relying solely on either time‐ or frequency‐domain analysis may overlook important correlations. Classifying the menstrual cycle only into follicular and luteal phases may result in assessments during periods of marked hormonal fluctuation, such as very low estrogen during early menstruation or peak estrogen around ovulation. Moreover, the use of the last menstrual period (LMP) to calculate and determine reproductive cycle phases, without accounting for normal cycle length variation that can occur even within the same individual, may lead to inaccurate phase classification and false interpretation of HRV‐hormone associations. Additionally, isolating the influence of each hormone through separate expression models neglects the combined effect of E2 and P4 on autonomic balance. In regard to these issues, accurate phase identification can be achieved through backward counting, which allows individualized cycle length calculation. Likewise, obtaining HRV recordings under carefully controlled conditions minimizes measurement error and ensures reliable assessment of both time‐domain and frequency‐domain parameters.

Therefore, this study aims to compare time‐domain and frequency‐domain HRV parameters across three more hormonally stable phases based on the physiological hormone levels in each phase: the pre‐ovulatory/menstrual phase, characterized by low estrogen and progesterone; the peri‐ovulatory phase, with high estrogen and low progesterone; and the premenstrual phase, during which both estrogen and progesterone are elevated. The Phases are determined by using individualized phase identification by backward counting in regard to cycle length, and to examine their associations with estradiol (E2), progesterone (P4), and the E/P ratio, with HRV measured under strictly controlled conditions to ensure reliability.

## 2 SUBJECTS AND METHODS

### 2.1 Study design, setting, and participant recruitment

This study followed a cross‐sectional observational design and was implemented among reproductive‐age females in Sulaymaniyah city, Iraq. Participants were enrolled through community‐based announcements via social media. Individuals expressing interest were thoroughly informed in their native language to ensure informed understanding of the study, inclusion criteria, and instructions. All volunteers provided written consent before participation. The confidentiality and anonymity of participants' responses and physiological data were ensured in accordance with General Data Protection Rules (GDPR). The research study received its ethical approval from the College of Medicine, University of Sulaimani, ethical committee, ID 309, 22‐9‐2024. It was conducted in accordance with the Declaration of Helsinki, and written informed consent was provided by participants.

Eligibility criteria included healthy females aged 18–45 years with regular menstrual cycles (defined as 21–35 days in length for at least 6 months) (Eunice Kennedy Shriver National Institute of Child Health and Human Development—NICHD, 2026; Hunt, 1993). Females were excluded from the study if they were pregnant, breastfeeding, or undergoing hormonal therapy (e.g., contraceptives or HRT), any medication affecting hormones or the ANS, any systemic disease or disease related hormonal disturbances, or with clinical record of chronic disease affecting autonomic function, besides females with any sign or symptom of menopausal transition or irregular cycle were excluded from the study.

### 2.2 Determination of menstrual cycle phases

Determining the menstrual cycle phase formed a key element in the study's analytical design. Data collection was aligned with three predefined menstrual cycle phases: menstruation/pre‐ovulatory (days 1–11), peri‐ovulatory (days 12–18), and pre‐menstrual (days 19–28), based on the highest level of E/P ratio, E2, and P4 in menstruation/pre‐ovulatory, peri‐ovulatory, and pre‐menstrual phases, respectively. Menstrual cycle phases were initially estimated using the first day of the last menstrual period (LMP) to provisionally assign participants to a cycle phase at the time of data collection. After completion of the cycle, participants reported the onset of their next menstruation, allowing determination of the exact cycle length. Final phase classification was then determined retrospectively by back‐extrapolation from the next menstruation date, assuming a relatively stable luteal phase of approximately 14 days to estimate the ovulation timing. The latter was finally selected due to its increased accuracy in identifying ovulatory and luteal events, providing the comparatively fixed duration of the luteal phase to improve phase classification of approximately 14 days.

Participants documented the start dates of their preceding and upcoming menstrual cycles. Final phase classification was identified through back‐extrapolation, allowing for accurate alignment of cycle days: for a 28‐day cycle, ovulatory phase (days −17 to −11), premenstrual phase (days −10 to −1), and menstruation/pre‐ovulatory phase (days −28 to −18). Proportional scaling is used for measuring the phases for each separate cycle duration from 21 to 35 days. A total of 32 participants were initially assigned to the pre‐ovulatory phase, 33 to the peri‐ovulatory phase, and 34 to the premenstrual phase.

### 2.3 Pre‐assessment protocol

Before physiological measurements, participants received a follow‐up briefing and were asked to confirm adherence with the pre‐assessment instructions, including abstention from intense physical activity, caffeine, and pharmacological agents influencing the autonomic nervous system. Assessments were rescheduled for those who had unintentionally consumed caffeine or did not comply with the protocol. Data collection was consistently scheduled between 08:30 and 11:30 to minimize diurnal variation. Data were collected from participants through the use of a structured interview guided by a general‐purpose questionnaire including sociodemographic characteristics. Anthropometric data were obtained objectively. Body weight was measured using an analytical scale, and height was measured using the stadiometer attached to the same device. Waist circumference was measured using a flexible tape measure at the midpoint between the iliac crest and the lower rib margin while participants were breathing normally (without breath holding). The waist/height ratio and BMI were subsequently calculated. These measurements were recorded by the investigators on the participant data form.

### 2.4 HRV measuring protocol (Schaffarczyk et al., 2022; Yang & Ben‐Menachem, 2024)

HRV was measured using the Polar H10 chest strap, paired with the Elite HRV software. Before recording, to minimize external influences, participants were instructed to remain seated at rest for 10–15 min in a calm environment. During data collection, participants were seated with their backs supported and feet flat on the ground. They were instructed not to move or speak and were asked to maintain natural breathing throughout the recording. The chest strap electrodes were lightly moistened for signal optimization, and positioning was adjusted until stable signal transmission was verified. Once signal stability and sensor placement were confirmed, 5‐min HRV recordings were obtained under controlled resting conditions. Parameters analyzed in the time domain included standard deviation of normal R‐R intervals (SDNN), root mean square of successive R‐R differences (RMSSD), mean RR interval in milliseconds (ms), low‐frequency power (LF), and high‐frequency power (HF) in their absolute value (ms^2^). SDNN was interpreted as an index of overall variability and balance between sympathetic and parasympathetic influences or overall HRV. The heart rate (HR) was also recorded along with HRV parameters. RMSSD and HF were primarily viewed as markers of parasympathetic tone, while LF reflects mixed sympathetic–parasympathetic modulation, though controversy exists in the literature (La Rovere et al., 2020; Pagani et al., 1997; Shaffer & Ginsberg, 2017). The LF/HF ratio has historically been interpreted as an index of sympathovagal balance (Pagani et al., 1997); however, this interpretation has been questioned, and the ratio should be interpreted cautiously as it may not reliably represent sympathetic–parasympathetic balance (Billman, 2013; Shaffer & Ginsberg, 2017).

### 2.5 Hormonal sampling and assay procedure

Following HRV measurement, a 5 mL venous blood sample was drawn. Blood samples were centrifuged (DLAB DM 0506) at 2500 rpm for 10 min within 1 h of collection. To minimize potential degranulation and ensure the same condition for all samples investigated, samples were aliquoted and stored at −80°C. After data collection had finished, hormone assays for E2 and P4 were conducted using the Cobas‐Elecsys‐2010 immunoassay analyzer. E2 concentrations were expressed in picograms per milliliter (pg/mL), while P4 was reported in nanograms per milliliter (ng/mL).

### 2.6 Statistical analysis

Data were systematically recorded into structured spreadsheets and subjected to a secondary review to ensure accuracy, data coding and cleaning were done, and then the data were imported into the statistical package of the social sciences (SPSS 26) software for statistical analysis. Normality tests and descriptive statistics are performed on all continuous variables; the normally distributed variables are shown as mean ± SD, which includes Age, BMI, waist circumference, waist/height ratio, HRV, mean RR interval, RMSSD, and SDNN. The non‐normally distributed variables are presented as median (interquartile range (IQR)). The categorical data are presented as frequencies and percentages. The comparison between groups is performed using parametric tests for the continuous variables; the ANOVA test for comparing the three menstrual group phases. While nonparametric tests, the Kruskal–Wallis H test is used for comparison between the three menstrual phase groups. The categorical data were compared using the chi‐square test. Spearman's correlations as a nonparametric test are used for finding correlation between the variables, and the Bonferroni correction is used to avoid type I error. Multiple linear regressions were computed for the HRV parameters as outcome variables.

## 3 RESULTS

Out of an initial pool of 112 women who enrolled voluntarily and completed phone‐based screening, 13 were excluded from analysis based on hormonal screening results, which indicated significantly abnormal E2 or P4 levels, 4 SD above the mean. Following this refinement, the final sample consisted of 99 women. After correcting for true cycle length adjustment from the next menstrual onset, revised classification groupings yielded 38, 30, and 31 participants in pre‐ovulatory/menstruation, peri‐ovulatory, and pre‐menstrual phases, respectively.

Among 99 analyzed individuals, the mean age is 30.5 ± 8.62 years with the age range of 18–45 years, and the mean BMI is 26.34 ± 4.78 Kg/m^2^. The mean of menstrual flow days is 5.85 ± 1.06 with an average cycle length of 28.41 days. The characteristics of the studied participants are presented in Table 1. The subjects were included according to the last menstrual period (LMP) and the group definition mentioned previously; 32 of them were in the menstruation/pre‐ovulatory phase, 33 in the peri‐ovulatory, and 34 in the pre‐menstrual phase. When the actual cycle length was calculated from their next period, the precise phase according to the menstrual phase definition was applied by back extrapolation. The number of subjects who lie within the menstruation/pre‐ovulatory, peri‐ovulatory, and pre‐menstrual are 38, 30, and 31, respectively.

**TABLE 1:** Baseline characteristics of the studied participants.

Parameters	Mean ± SD
	Frequency (%)
	*n* = 99
Age (y)	30.5 ± 8.62
BMI (Kg/cm^2^)	26.34 ± 4.78
Waist circumference (cm)	81.37 ± 9.50
Waist/Height ratio	0.52 ± 0.08
Menstrual flow duration (days)	5.9 ± 1.1
Average cycle length (days)	28.4 ± 3.2
Actual cycle length (days)	29.9 ± 3.9
Menstrual phases^a^	
Pre‐ovulatory phase (Menstruation)	38 (38.38%)
Per‐ovulatory phase	30 (30.31%)
Pre‐menstrual phase	31 (31.31%)

*Note*: Continuous variables are shown as mean ± SD.

Abbreviation: BMI, body mass index.

^a^ Categorical variables are shown as frequency (%).

The comparisons of all variables across the three menstrual phase groups of the total participants are shown in Table 2. There are no significant differences in age, anthropometric measures, and HR between different menstrual phase groups.

**TABLE 2:** Comparison of ovarian hormones and HRV parameters across menstrual phase groups.

Parameters		Menstruation/pre‐ovulatory (*n* = 38)	Peri‐ovulatory (*n* = 30)	Pre‐menstrual (*n* = 31)	*p* Value
		Mean (SD)	Mean (SD)	Mean (SD)	
		Median [range]	Median [range]	Median [range]	
Sociodemographic and anthropometric	Age (y)*	29.7 (8.6)	31.4 (9.2)	29.2 (8.3)	0.58
	BMI (kg/cm^2^)*	26.9 (5.3)	25.7 (4.8)	26.3 (4.2)	0.59
	Waist circumference (cm)*	82.5 (10.7)	80.7 (9.8)	80.6 (7.6)	0.63
	Waist/Height Ratio*	0.538 (0.114)	0.506 (0.065)	0.512 (0.047)	0.23
Hormonal	Estradiol (pg/mL)				
	Mean	66.3 (40.7)	153.2 (112.4)	134.9 (77.0)	**<0.001**
	Median	48 [13.6–154.6]	109 [39.5–585.0]	130 [17.6–330.7]	
	Progesterone (ng/mL)				
	Mean	1.07 (2.6)	4.43 (4.0)	8.27 (6.6)	**<0.001**
	Median	0.28 [0.16–12.91]	2.70 [0.10–12.22]	7.66 [0.15–20.39]	
	E/P ratio	25.0 [9–100.2]	17.5 [8.0–85.0]	16.0 [7.0–80.0]	**0.04**
Heart rate and heart rate variability Time domain Frequency domain	HR (bpm)*	81.2 (10.4)	79.1 (7.8)	83.8 (9.5)	0.11
	RMSSD (ms)*	29.5 (17.2)	36.2 (18.2)	29.3 (13.8)	0.19
	SDNN (ms)*	40.2 (13.9)	47.7 (15.8)	43.5 (12.7)	0.07
	Mean RR interval (ms)*	751 (92)	774 (86)	725 (78)	0.05
	LF (ms^2^)	429 [55–1622]	399 [146–1622]	409 [150–1350]	0.79
	HF (ms^2^)	217 [19–2119]	397 [46–2269]	243 [25–3240]	**0.03**
	LF/HF ratio	1.6 [0.4–8.1]	1.0 [0.4–6.6]	1.7 [0.3–22.5]	0.39

*Note*: Non‐normally distributed variables are presented as median [interquartile range] and compared using the Kruskal–Wallis test. Estradiol and progesterone are also presented as mean (SD), although statistical comparisons were performed using median [IQR]. Variables with normal distribution are indicated with an asterisk (*) and are presented as mean (SD) and compared using one‐way ANOVA. Statistically significant *p* values (*p* < 0.05) are shown in bold.

Abbreviations: HF, high‐frequency power; LF, low‐frequency power; RMSSD: root mean square of successive R‐R differences; SDNN, standard deviation of normal R‐R intervals.

**FIGURE 1:**
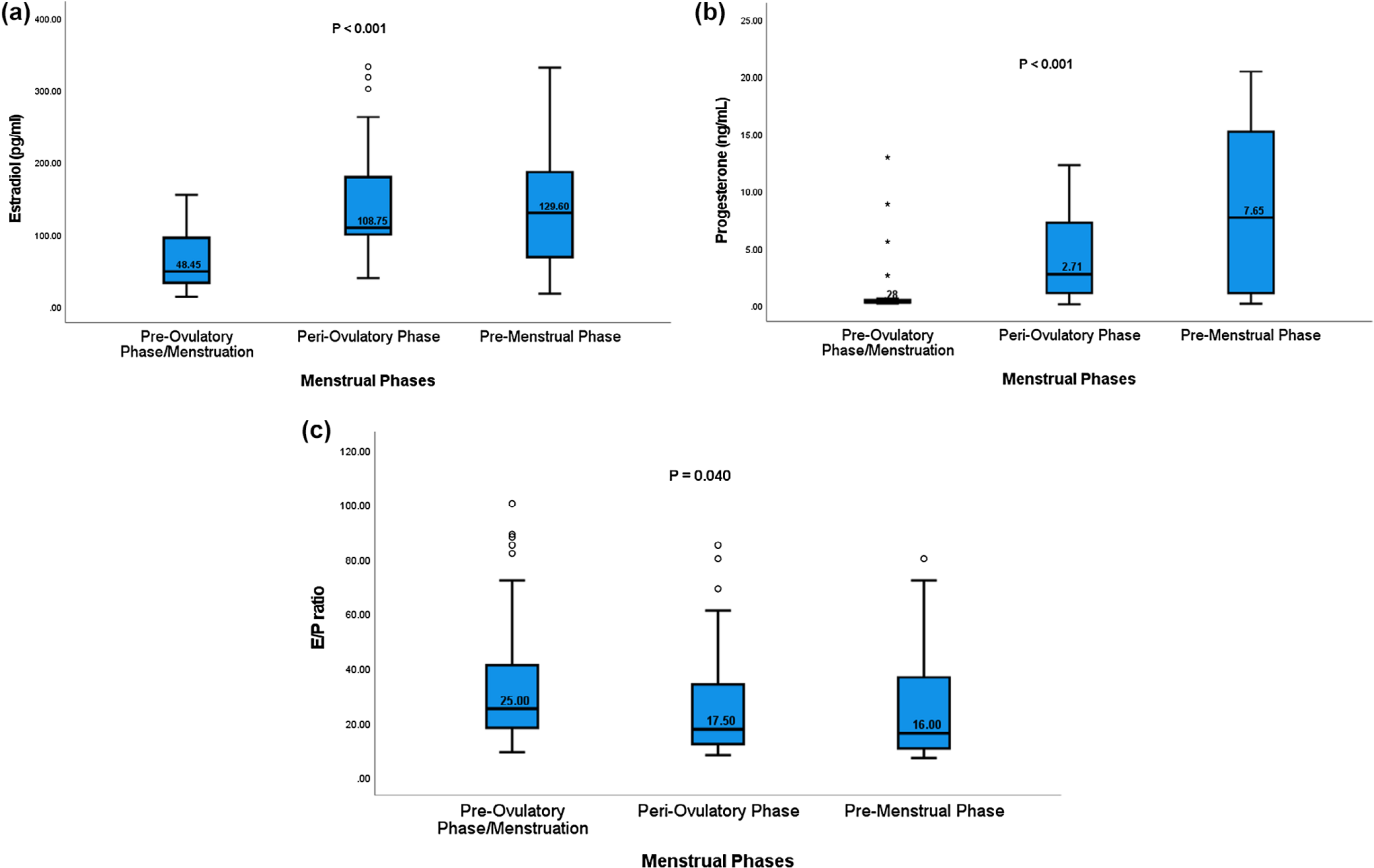
(a–c) Average progesterone (a), estradiol (b), and E/P ratio (c) between different menstrual phase groups. Data are presented as box‐and‐whisker plots. The box represents the interquartile range (IQR), the line within the box indicates the median, and the whiskers represent the minimum and maximum values.

Mean serum E2 and P4 levels are highest at peri‐ovulatory and pre‐menstrual phases, respectively, while the E/P ratio is highest at menstruation/pre‐ovulatory phase with statistically significant results, Figure 1 and Table 2.

**FIGURE 2:**
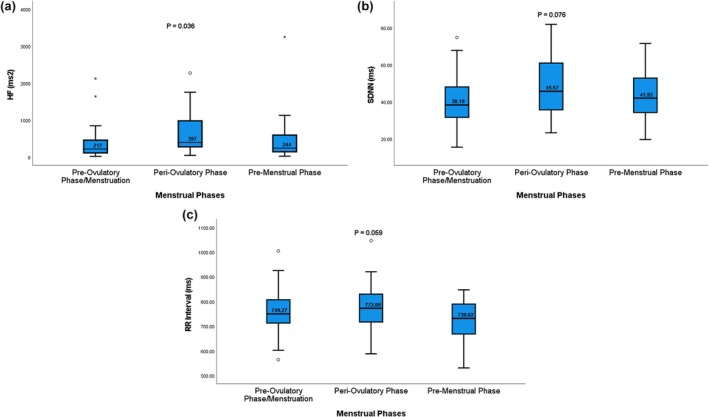
(a–c) Average HF (a), SDNN (b), and mean RR intervals (c) between different menstrual phase groups. Data are presented as box‐and‐whisker plots. The box represents the interquartile range (IQR), the line within the box indicates the median, and the whiskers represent the minimum and maximum values.

Evaluation of the HRV parameters reveals higher HF, SDNN, and mean‐RR intervals in the Peri‐ovulatory phase compared to the other phases, with only HF reaching the statistically significant level, *p* = 0.036, 0.076, and 0.059, respectively (Figure 2).

**FIGURE 3:**
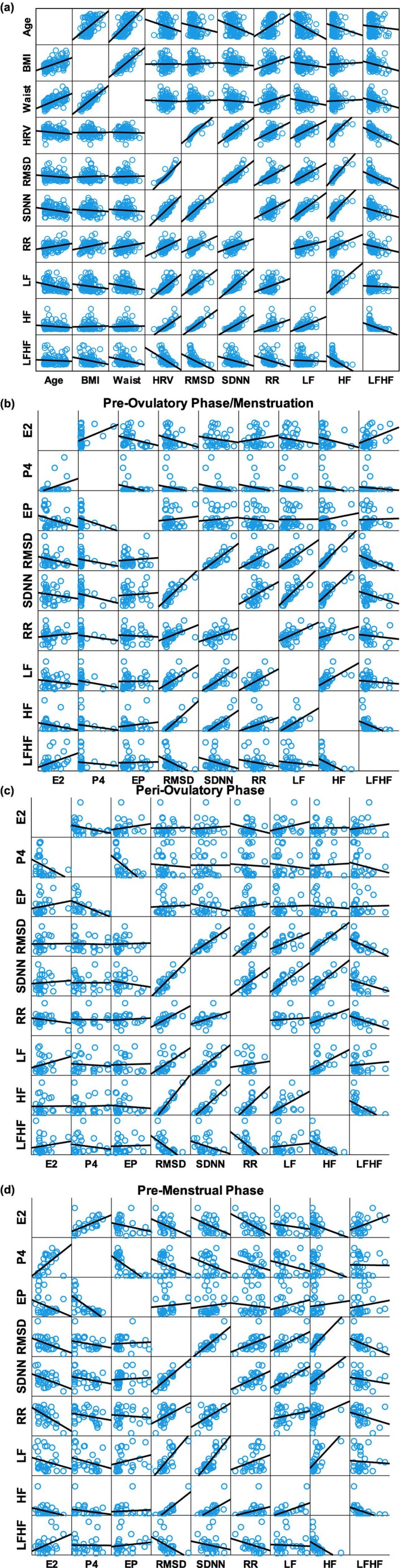
(a–d) Linear correlation of HRV parameters with anthropometric measures (a) and ovarian hormones across the three menstrual phases (b–d) in studied participants. Each blue circle represents a bin that contains several participants, and the size of the circles indicates how many observations fall in that bin. The numbers indicate the approximate number of observations that fall in that bin.

Linear correlations between age, BMI, waist circumference, and HRV parameters in all participants are shown in Figure 3. Age was negatively correlated with most HRV parameters, except for the mean RR‐interval, which showed a positive correlation, and these correlations remained significant even after Bonferroni correction (SDNN: ρ = −0.263, *p* value = 0.007, LF ρ = −0.374, *p* value < 0.001, mean RR‐interval: *ρ* = 0.314, *p* value = 0.001). Mean RR‐interval was also positively correlated with BMI and waist circumference. Another significant correlation was observed between waist circumference and LF/HF (*ρ* = −0.262, *p* value 0.007). Linear correlation between hormonal and HRV parameters across different phase groups is also presented in Figure 3. In the menstruation/pre‐ovulatory phase group, E2 is negatively correlated with the HF (ρ = −0.338, *p* value = 0.035), but does not reach a statistically significant value after Bonferroni correction, which required a *p* value of < 0.008. In the peri‐ovulatory stage, no significant correlation is detected between parameters. In the pre‐menstrual phase group, the significant correlation includes the negative correlation between E2 and all HRV parameters, and positive correlation between EP and LF (ρ = 0.406, *p* value = 0.024), while the only significant correlation remaining after Bonferroni correction is the negative correlation between E2 and mean RR‐intervals (ρ = 0.515, *p* value = 0.003).

The only significant independent predictor of HRV (adjusted R‐squared = 0.283, *p* = 0.010) is E2 at the premenstrual phase, which negatively predicts mean RR‐interval and BMI at the peri‐ovulatory phase when simple linear regressions were analyzed for each of the HRV parameters separately as dependent variables (Table 3). None of the variables were regarded as significant predictors of HRV parameters in the pre‐ovulatory phase, and only BMI at the peri‐ovulatory phase.

**TABLE 3:** Multiple linear regression analysis for HRV parameters as dependent variables at menstrual phases.

Model	Unstandardized coefficients		Standardized coefficients	*p* Value	95% CI for B	
	B	SE	Beta		Lower bound upper bound	
Peri‐menstrual phase	Dependent: mean RR‐interval					
BMI	13.321	4.97	0.73	**0.013**	3.052	23.59
Pre‐menstrual phase	Dependent: mean RR‐interval					
Estradiol	−0.450	0.162	−0.456	**0.010**	−0.782	−0.118

*Note*: Only predictors that remained statistically significant in the final multivariable regression model are presented. Statistically significant *p* values (*p* < 0.05) are shown in bold.

Abbreviations: B, beta coefficient; CI, confidence intervals; SE, standard error of mean.

## 4 DISCUSSION

This study underscored the complexity of phase‐specific associations between sex hormones and HRV parameters in reproductive females. HRV markers such as HF peaked during the peri‐ovulatory phase, suggesting enhanced parasympathetic activity during hormonally unstable intervals with greatly elevated E2 levels. This is in agreement with previous studies, which observed a reduction in HF from the follicular to the luteal phase. Regarding HR, although a previous study reported a drop in HR before ovulation (Biswas & Ghosh, 2024), no significant changes in HR were observed between menstrual phases in the present study. This discrepancy may be related to differences in study design, sample characteristics, or measurement conditions.

In the current study, although age has a correlation with HRV parameters in reproductive females, it does not predict any HRV parameters, while BMI significantly predicts HRV (mean RR‐interval) at the peri‐ovulatory phase. In a multivariable analysis, studying the effect of age, BMI, and menstrual cycle on HRV, they found age to a first predictor for HRV, followed by BMI and menstrual cycle. They found that HRV was higher in younger females with lower BMI at the follicular phase (Vallejo et al., 2005).

The current study demonstrates E2 as an independent negative predictor of HRV parameters in the premenstrual phase, while no significant correlation between P4 and HRV parameters was found throughout the reproductive cycle. Inconsistent with the current study results, in some of the previous studies, P4 was found to be a key hormonal driver of autonomic regulation throughout the cycle, and it displayed a consistent inverse relationship with vagal tone. In contrast, E2 did not exhibit significant effects in any phase in a few studies (Saperova & Filippova, 2022; Sato et al., 1995; Schmalenberger et al., 2020), while E2 correlated positively with parasympathetic activity in other studies (Biswas & Ghosh, 2024; Leicht et al., 2003; Robles‐Cabrera et al., 2025). Discrepancies between the aforementioned findings (Robles‐Cabrera et al., 2025; Saperova & Filippova, 2022; Schmalenberger et al., 2020) and the current study are likely attributed to the methodological approach, using different samples, such as salivary (Schmalenberger et al., 2020) or urinary hormone assay (Saperova & Filippova, 2022). The current study's observation raises the possibility that E2's effects may be modulated by P4 during most of the cycle but become more pronounced during hormonal fluctuation periods late in the luteal phase, notably when P4 begins to fall after an initial rise. Alternatively, rapid changes in hormonal levels may be responsible rather than absolute hormone concentrations. Progesterone levels in some participants appeared as outliers in the box plot, indicating higher values compared with the overall distribution and likely reflecting individual variability in hormone levels.

Although it does not reach a statistically significant value, there is a correlation between LF and E/P ratio in the present study, in partial agreement with observations in Bai et al. study (Bai et al., 2009), which reported a significant correlation between a HRV measure and E2/P4 ratio, hinting that it is not the absolute value of either hormone, but rather their relative balance, that might shape autonomic output. In a cross‐sectional study of 50 reproductive women, higher mean RR‐intervals and other HRV parameters were found in the secretory compared to the menstruation phase, and they concluded parasympathetic dominance during the proliferative phase in comparison to sympathetic dominance of the secretory phase (Brar et al., 2015). A study on 19 active females assessing their physical activity with their HRV throughout menstrual phases reveals the same finding (Pestana et al., 2018). Another study (Saperova & Filippova, 2022) likewise adds meaningful perspective to the findings: it found notable phase differences in HRV parameters, with elevated HF and SDNN in the follicular phase and higher stress markers in the luteal phase.

Providing further evidence for the sympathetic dominance in late‐cycle phases, Matsumoto et al. (2006) found that women presenting with marked premenstrual symptoms exhibited elevated LF/HF ratios and reduced parasympathetic activity. These effects were evident only in the symptomatic subgroup and only during the luteal phase. While Chung and Yang (2011), found no evidence of significant correlations between E2 and HRV parameters, it is worth noting that this study was conducted among shift‐working nurses, and HRV measurements were taken during sleep periods. A research context in which circadian misalignment may act as a confounding variable (Chung & Yang, 2011). Similarly, Sato et al. (1995) confirmed increased LF/HF and reduced HF in the luteal phase, but did not analyze hormonal predictors, highlighting a gap that the current study addresses.

Finally, the nonlinear parameters from Bai et al. (2009) introduce a new perspective, suggesting that the E2/P4 balance, rather than absolute E2 levels, may more accurately capture autonomic fluctuations. This supports the current study's implication that hormonal ratios may offer greater explanatory value for HRV variability compared to analyzing absolute hormone concentrations in isolation, particularly at cycle extremes like the premenstrual phase.

While much of the literature (Robles‐Cabrera et al., 2025; Saperova & Filippova, 2022; Sato et al., 1995; Schmalenberger et al., 2019) regarded P4 and E2 as negative and positive key modulators of HRV, respectively, the current findings refine the existing view by identifying an opposing role for E2, which is negatively correlated with HRV at pre‐menstrual, that is phase‐specific, and its correlation with the time domain rather than the frequency domain is statistically significant. The differences between the current study's findings and previous reports may be attributable to methodological variations. For example, in the study by Robles‐Cabrera et al. (2025); HRV was primarily examined across different body positions within menstrual cycle phases, rather than comparing HRV between phases themselves. In addition, HRV measurements were obtained while participants were in standing or supine positions, whereas measurements in the present study were performed in the sitting position. Furthermore, their study included postmenopausal women and did not apply the same three‐phase menstrual cycle classification used in the present study. These findings support the importance of a menstrual phase‐specific approach to hormone‐HRV analysis, acknowledging that the autonomic response to hormonal fluctuation varies not only by a single hormone, but also by a combination of hormones or a rapid hormonal fluctuation across the cycle.

Several methodological limitations are present in many previous studies (Armbruster et al., 2018; Leicht et al., 2003; Matsumoto et al., 2006; Weissman et al., 2009). These include simplified classification of the menstrual cycle into only follicular and luteal phases, without accounting for hormonal differences within the follicular phase and reliance solely on the last menstrual period to determine cycle phase without considering individual cycle‐length variability. In addition, many studies assessed only selected HRV parameters, frequently focusing on either frequency‐ or time‐domain indices rather than a complete HRV profile. Furthermore, evaluating hormones independently may overlook the interactive effects of estradiol (E2) and progesterone (P4) on autonomic regulation (Armbruster et al., 2018; Matsumoto et al., 2006). Most studies have often examined the isolated effects of E2 or P4 on autonomic function or evaluated HRV across menstrual phases without considering hormonal interactions. Such approaches may overlook the combined or dynamic effects of multiple hormones, which may contribute to inconsistent findings in the literature.

The present study addressed several of these limitations. The menstrual cycle was divided into three hormonally distinct phases, with phase identification performed using backward counting from the subsequent menstruation to account for individual cycle length. HRV measurements were collected at standardized time points, and both time‐ and frequency‐domain parameters were analyzed. Participants were also instructed to avoid behaviors known to influence HRV before assessment, minimizing potential confounding and strengthening the reliability of the findings. In the present study, the peri‐ovulatory phase, characterized by elevated E2 levels, was associated with increased parasympathetic activity. However, this pattern differed in the premenstrual phase, where E2 demonstrated a negative association with HRV. These findings suggest that parasympathetic modulation during the menstrual cycle cannot be attributed solely to estradiol levels and may also involve interactions with other hormones or rapid hormonal fluctuations.

One of the limitations of this study is that HRV and hormone levels were not repeatedly measured across all phases within the same participants. Future studies should incorporate repeated hormonal measurements and include additional hormones, such as luteinizing hormone (LH), follicle‐stimulating hormone (FSH), testosterone, and cortisol to better characterize the complex and phase‐dependent relationship between hormonal fluctuations and autonomic regulation. From a clinical perspective, hormonal fluctuations across the menstrual cycle may influence cardiac electrophysiology. Variations in QT interval duration and susceptibility to supraventricular tachycardia (SVT) have been reported across menstrual phases, with increased arrhythmic events occurring in the premenstrual period. These findings highlight the importance of considering menstrual cycle phase in cardiovascular assessment in women (Schreuder et al., 2019).

In conclusion, HRV varies significantly across menstrual cycle phases, with parasympathetic activity, as reflected by HF, being highest during the peri‐ovulatory phase. Additionally, the mean RR interval demonstrated a significant negative association with estradiol levels. These findings suggest that autonomic regulation during the menstrual cycle reflects a complex, phase‐dependent interaction between reproductive hormones rather than the effect of a single hormone.

## AUTHOR CONTRIBUTIONS

**Tara R. Salih:** Data curation; investigation; methodology; resources; software; validation. **Darya S. Abdulateef:** Conceptualization; formal analysis; methodology; project administration; resources; software; supervision; validation; visualization.

## FUNDING INFORMATION

This research did not receive any specific grants from funding agencies in the public, commercial, or not‐for‐profit sectors.

## CONFLICT OF INTEREST STATEMENT

The authors declare no conflicts of interest and have received no funding or grants for this study.

## ETHICS STATEMENT

The research study received its ethical approval from College of Medicine, University of Sulaimani ethical committee, ID 309, 22‐9‐2024. It was conducted in accordance with the Declaration of Helsinki, and written informed consent was provided by participants.

## Data Availability

The data used in this study are available in the figshare data repository, and the link will be provided by the corresponding author upon reasonable request.
